# Health-Related Stigma as a Determinant of Functioning in Young Adults with Narcolepsy

**DOI:** 10.1371/journal.pone.0122478

**Published:** 2015-04-21

**Authors:** Mary C. Kapella, Barbara E. Berger, Boris A. Vern, Sachin Vispute, Bharati Prasad, David W. Carley

**Affiliations:** 1 Department of Biobehavioral Health Science, Center for Narcolepsy, Sleep & Health Research, College of Nursing, University of Illinois at Chicago, Chicago, Illinois, United States of America; 2 Department of Pulmonary, Critical Care, Sleep & Allergy, College of Medicine, University of Illinois at Chicago, Chicago, Illinois, United States of America; Hospital General Dr. Manuel Gea González, MEXICO

## Abstract

Symptoms of narcolepsy tend to arise during adolescence or young adulthood, a formative time in human development during which people are usually completing their education and launching a career. Little is known about the impact of narcolepsy on the social aspects of health-related quality of life in young adults. The purpose of this study was to examine relationships between health-related stigma, mood (anxiety and depression) and daytime functioning in young adults with narcolepsy compared to those without narcolepsy. Young adults (age 18–35) with narcolepsy (N = 122) and without narcolepsy (N = 93) were mailed a packet that included questionnaires and a self-addressed postage paid envelope. The questionnaire included demographic information and a composite of instruments including the SF 36, Functional Outcomes of Sleep Questionnaire (FOSQ), Fife Stigma Scale (FSS), Epworth Sleepiness Scale (ESS) and Hospital Anxiety and Depression Scale (HADS). Variable associations were assessed using descriptive statistics, ANOVA, Mann-Whitney U Test, correlations, stepwise multiple regression and path analysis. Young adults with narcolepsy perceived significantly more stigma and lower mood and health-related quality of life than young adults without narcolepsy (p<0.01). Health-related stigma was directly and indirectly associated with lower functioning through depressed mood. Fifty-two percent of the variance in functioning was explained by the final model in the young adults with narcolepsy. Health-related stigma in young adults with narcolepsy is at a level consistent with other chronic medical illnesses. Health-related stigma may be an important determinant of functioning in young adults with narcolepsy. Future work is indicated toward further characterizing stigma and developing interventions that address various domains of stigma in people with narcolepsy.

## Introduction

Health-related stigma is defined by Weiss and colleagues[1] as “a social process, experienced or anticipated, characterized by exclusion, rejection, blame or devaluation that results from experience, perception or reasonable anticipation of an adverse social judgment about a person or group”. Health-related stigma has been reported in a number of chronic illnesses, including narcolepsy[2] and is identified as a potential predictor of lower health-related quality of life (HRQOL) and health disparities[3]. Health-related stigma has been associated with lower quality of life in people with chronic illnesses such as Parkinson’s disease[4,5] and epilepsy[6], but has yet to be examined in people with narcolepsy.

Narcolepsy is a chronic, incurable neurologic disorder characterized by some or all of the following symptoms, in order of frequency: excessive daytime sleepiness (EDS), cataplexy, hallucinations upon awakening or going to sleep, sleep paralysis, and disturbed nighttime sleep[4,5]. Among these symptoms, EDS and cataplexy usually present the greatest challenge to the patient and treating physician alike. Medical treatment includes drugs which: (1) suppress the EDS (amphetamines; modafinil/armodafinil; sodium oxybate); and (2) suppress cataplexy and sleep paralysis (sodium oxybate; antidepressants).

Whereas the age range of onset of many chronic medical conditions such as mental illness, physical disability and HIV/AIDS is variable, narcolepsy is notable for an overall bimodal temporal pattern of onset, with the major peak at about 15 years and a minor one at 35 years[6]. The post-adolescent through young adulthood period is an important formative time during which people are not only preparing for and launching a career through a successful educational program, but are also acquiring the self-confidence and skills necessary for an ultimately effective and satisfying social integration. However, despite the usually early onset of the signs of narcolepsy, some individuals may remain symptomatic for 20 years or more before a correct diagnosis and appropriate treatment are achieved, despite repeated encounters with different health care providers[5]. Thus, the young adult with narcolepsy may become stigmatized in one of two ways: because of the absence of a medical explanation for the disruptive episodes of sleepiness, or because of the confirmed presence of a diagnosis that itself may generate stigmatization.

Health-related stigma has the potential to limit healthy psychosocial development in a number of important areas. Studies have reported low health-related quality of life in people with narcolepsy[7–13], but most of what is known comes from surveys of adults over a wide range of ages or who are middle-aged or older. Marital difficulties are common[14] and depression frequently occurs[11,14]. Recent studies of adults in their 30’s[15,16] reported low health-related quality of life in younger adult narcolepsy patients with depression and occupational and academic difficulties including deleterious effects on personal and social relations. Patients diagnosed earlier perceived their health as better, attained higher educational level and had less employment problems than those diagnosed later in life[16].

While there is, therefore, considerable evidence of low health-related quality of life in adults with narcolepsy, the actual underlying mechanisms contributing to it have yet to be fully defined. Young adults with narcolepsy have reported feeling set apart (even by members of their own family), inferior to others because of their disorder symptoms, and hesitant to disclose their disorder to others because of fears about the consequences and reaction they would receive[17]. Given the intensive symptoms of narcolepsy that are often difficult to manage, health-related stigma associated with narcolepsy is likely to have a negative impact on the quality of life of young adults and is likely partially responsible for the depression, occupational and academic difficulties and personal and social problems reported by younger patients.

The present study builds upon previous research to provide a better understanding of the impact of stigma and related variables associated with HRQOL in young adults with narcolepsy. We sought to compare young adults with and without narcolepsy on health-related stigma, mood and health-related quality of life, determine relationships among the variables and identify predictors of functioning in young adults with narcolepsy. Results provide evidence for further research on stigma and the development of interventions for people with narcolepsy.

## Materials and Methods

### Design & Sample

For this cross sectional survey study we utilized data collected by Merritt and colleagues in 2002[18]. The sample consisted of young adults with narcolepsy 18–37 years of age who contacted the University of Illinois at Chicago, Center for Narcolepsy Research asking that they be placed on the mailing list and indicating an interest in participating in research. An acquaintance approach was used to obtain a control group of young adults without narcolepsy[19]. Young adults with narcolepsy answering an advertisement by phone and agreeing to participate were mailed a packet that included the questionnaire along with a cover letter and a self-addressed postage paid envelope. The questionnaire included demographic, socioeconomic and disease-related information and a composite of instruments including the Short Form 36[20], the modified Social Impact Scale (MSIS)[21], the Hospital Anxiety and Depression Scale (HADS)[22,23], the Epworth Sleepiness Scale (ESS)[24] and the Pittsburgh Sleep Quality Index[25].

### Ethics Statement

The principal investigator provided information In the cover letter explaining the purpose and procedures of the study and confidentiality information generally found in the consent form. Also included in the cover letter was a statement that participation in the study was voluntary and that by returning the completed survey, the subject provided consent to be included in the study. Survey data were anonymized and de-identified. The study was approved by the University of Illinois at Chicago Institutional Review Board.

### Measures

Health-related stigma was measured using the Stigma and Social Impact Scale (SSIS)[21] and the Disclosure Concerns scale[26] The SSIS includes 24 items with a 4-point likert response scale (strongly agree, agree, disagree and strongly disagree). It consists of four subscales: social rejection (9 items), financial insecurity (3 items), internalized shame (5 items) and social isolation (7 items) experienced in the past 4 weeks. Social rejection signifies the feelings of social and job rejection experienced by the respondent. Financial insecurity captures feelings of the financial consequences of discrimination including income and job security. Internalized shame indicates the extent to which the participant has feelings of being different from others, blames his/her self for the illness and feels the need to conceal the illness. Social isolation captures feelings of low self-esteem and loneliness[21]. The items were reworded on the control version to focus the item on the condition of the respondent’s health. Scores were reversed so that higher scores reflect greater health-related stigma, and summed to calculate the subscale scores. The Cronbach’s alphas for the Stigma social rejection, financial insecurity, internalized shame and social isolation scales in this study were. 91,. 74,. 79 and. 93 respectively. Concern about disclosing health conditions to others was measured using the 11-item Disclosure Concerns scale (DC) developed by Berger[26]. The response scale of the DC was the same as the SSIS. Reliability and validity was supported in HIV patients[26] and Cronbach alpha in this study was. 88.

The well-validated and extensively utilized Short Form Health Survey (SF-36) [19,25] was used to measure health-related quality of life. The 36-item survey was constructed for self-administration by people 14 years of age or older. Items are rated on a likert scale and the instrument consists of 8 scales measuring facets of health-related quality of life: physical functioning, role limitations due to physical problems, bodily pain, vitality, general health perceptions, social functioning, role limitations due to emotional problems, and mental health[20]. Scores for the 8 scales were converted into a USA norm-based score, a standardized t score transformation (mean = 50 ± 10) that ranged from 0 to 100 with higher scores reflecting perceptions of better health. The Cronbach’s alphas for the SF-36 scales in this study ranged from. 81 to. 92.

The well-validated Functional Outcomes of Sleep Questionnaire (FOSQ)[27,28] was used to measure sleepiness-related functioning in the young adults with narcolepsy. This 30-item instrument is disease-specific and designed to assess the impact of disorders of excessive sleepiness on multiple activities of everyday living. Difficulty with functioning is rated on a 5-point Likert scale with 0 = no difficulty to 4 = extreme difficulty. The instrument includes 5 subscales: activity level, vigilance, intimacy and sexual relationships, general productivity, and social outcome. The Cronbach’s alpha for the total FOSQ in this study was. 89.

Anxiety and depression were assessed using the Hospital Anxiety and Depression Scale (HADS) [22,23], a well-validated instrument for detecting states of anxiety and depression.The HADS includes 14 items rated on a four point Likert scale. Higher scores reflect greater anxiety or depression. Scores for each subscale (anxiety and depression) can range from 0–21 with normal = (0–7), mild = (8–10), moderate = (11–14), severe = (15–21)[29]. The Cronbach’s alphas for the HADS anxiety and depression scales in this study were. 81 and. 85 respectively.

The Epworth Sleepiness Scale (ESS) [24] was used to measure the severity of daytime sleepiness. Respondents rated eight items regarding the likelihood of dozing in sedentary situations on a scale from 0 (never) to 3 (high chance). The Cronbach’s alpha for the ESS in this study was. 90.

Nighttime sleep quality was measured by the well-validated Pittsburgh Sleep Quality Index (PSQI)[25]. This 24-item instrument measures subjective sleep quality with a Global Sleep Quality Index (the sum of seven component scores). Higher scores indicate worse sleep quality. A Global Sleep Quality Index greater than 5 indicates poor sleep quality and difficulties with sleep in at least two areas. Reliability and validity were demonstrated in several chronic disease populations[30]. The Cronbach’s alpha for the PSQI in this study was. 74.

Data on demographic characteristics of age, gender, race, marital status, education, employment status (including whether employed and if so what shift worked), and narcolepsy-related information regarding symptoms (excessive daytime sleepiness, sleep attacks, cataplexy, hypnagogic hallucinations and sleep paralysis). Medication information and time from symptoms to diagnosis were also collected.

### Statistical Analysis

Analyses were conducted with IBM SPSS, version 21 (IBM Corp., 2012). Item missing values were replaced using mean substitution. For unit missing data, we determined whether missing data were MCAR (missing completely at random). Missing data occurred randomly. Data were analyzed with descriptive statistics and analysis of variance (ANOVA) was used to compare sample characteristics between groups. Bivariate relationships between key variables were examined with Spearman’s and Pearson correlations. Mann-Whitney U Test was used to compare stigma and HRQOL variables between groups. Hierarchical multiple regression using the “enter” method was employed to identify predictors of the total FOSQ in young adults with narcolepsy. The assumption of normaility of residuals was assessed by a Q-Q plot and variance inflation factors were assessed and found to be < 5. Independent variables were chosen from those found to be associated with HRQOL in previous research and those most significantly correlated with HRQOL in our data.

Simultaneous relationships among variables were tested using path analysis with IBM SPSS AMOS 22 software. The sample size (122) allowed for 9.4 subjects per parameters to be estimated in the path analysis. Statistical assumptions of normality, linearity, and homoscedasticity were tested, and necessary assumptions were met. The fit of the hypothesized model was tested using maximum likelihood estimation. The path model was refined by removing non-significant variables until the theoretically based model with the best fit was determined. Several fit indices were used to evaluate the model fit: chi square, normed fit index (NFI), comparative fit index (CFI), and the root mean square error of approximation (RMSEA).

## Results

The sample consisted of 122 young adults with narcolepsy and 93 young adults without narcolepsy. Sample characteristics are shown in Table 1. Participant ages ranged from 18 to 37 years with a mean age of 27 in the narcoleptics and 26 in the controls. The narcoleptics were slightly older and less educated, although both groups were fairly educated. There were more women than men and most participants were white. More than half were married or in a committed relationship and reported some college education.

**Table 1:** Sample characteristics.

Characteristics	Narcolepsy (n = 122)	Control (n = 93)	*P* value
Age (range = 18–35)	27.1 ± 5	25.7 ± 4	0.02
Female (%)	77.9	63.4	0.02
Race/ethnicity (%)			0.02
American Indian/Alaskan	1.6	1.1	
Asian	0.8	10.8	
Black	3.3	3.2	
Hispanic/Latino	2.5	4.3	
White/Non-Hispanic	87.7	79.6	
Other	4.1	1.1	
Educational status (%)			0.002
Some high school, high school or vocational	24.6	17.2	
Some College, College, and greater than college	75.4	82.8	
Student (%)	30.3	46.2	0.02
Marital status (%)			0.89
Married or Committed relationship	45.9	48.4	
Single	49.2	47.3	
Divorced/separated	4.9	4.3	
Employment (%)			
Employed	82.8	95.7	0.003
On sick leave	1.6	0.0	
Laid off	0.8	0.0	
On disability	9.8	0.0	
Homemaker	9.8	3.2	
Employment status of those working (%)			>0.05
Works ≤ 20 hours per week	9.0	11.8	
Works 21–35 hours per week	11.5	12.9	
Works ≥ 36 hours per week	41.8	41.9	
Previously discharged from a job (%)	33.0	14.1	0.02

Analyses are reported as mean (+/- SD) for continuous variables and percentages for

categorical variables.

Eighty-four percent of the participants with narcolepsy reported cataplexy. They indicated (mean ± SD) 4.8 ± 5 years between noticing symptoms of narcolepsy and obtaining the diagnosis of narcolepsy and 5.3 ± 4 years from diagnosis to date of data collection for this study. Ninety-five percent of the narcoleptics were taking wake-promoting medications, 47% were taking anti-depressants, 34% were taking anti-anxiety medications and 2% were taking sleep-promoting medications at bedtime. Medications were not associated with the total FOSQ score (r = -.12 to. 06, *p>*.*20*). Their mean total narcolepsy symptom count of 154 ranged from a minimum of 56 to maximum 346.

Most participants were employed but narcoleptics were less employed than controls. More than 12% of narcoleptics were on sick leave, laid off or on disability, versus none of the controls. Over 30% of the narcoleptics reported that they had previously been discharged from a job—significantly more than the controls. Fifty-four percent of participants with narcolepsy worked the day shift, 7% worked evenings, 2% worked nights and 8% worked rotating shifts. There was no difference between groups on the hours worked per week. Forty-two percent of working narcoleptics worked more than 35 hours per week and 30% were students.

Descriptive statistics for the key variables are shown in Table 2. There were significant differences between groups on all domains of health-related stigma and quality of ilfe and functional status, anxiety, depression, daytime sleepiness and nighttime sleep quality.

**Table 2:** Descriptive statistics: Key variables.

Characteristics	Narcolepsy (n = 122)	Control (n = 93)	Mann-Whitney U*P* value
Perceived Stigma (SSIS) Total Score	52.3 ± 14.4	30.9 ± 10.5	<0.001
Social Rejection	17.8 ± 5.8	10.7 ± 3.4	<0.001
Financial Insecurity	7.3 ± 2.8	4.1 ± 1.8	<0.001
Internalized Shame	10.2 ± 3.4	7.0 ± 2.8	<0.001
Social Isolation	17.1 ± 5.3	9.2 ± 3.7	<0.001
Disclosure Concerns	23.8 ± 7.7	15.6 ± 5.8	<0.001
HADS Anxiety	8.2 ± 4.3	6.7 ± 3.9	0.011
HADS Depression	7.1 ± 4.4	3.2 ± 2.9	<0.001
SF36 QOL (norm-based)			
Physical Function (PF)	49.2 ± 10.4	54.9 ± 4.0	<0.001
Bodily Pain (BP)	49.3 ± 10.9	53.1 ± 6.6	0.027
Role Physical (RP)	39.5 ± 10.4	53.5 ± 6.1	<0.001
General Health (GH)	43.8 ± 10.7	52.4 ± 8.4	<0.001
Vitality (V)	37.0 ± 8.7	48.2 ± 7.6	<0.001
Social Functioning (SF)	36.5 ± 13.7	49.7 ± 7.6	<0.001
Role Emotional (RE)	42.4 ± 12.8	47.1 ± 11.3	0.006
Mental Health (MH)	42.4 ± 10.9	47.5 ± 8.3	<0.001
FOSQ Total Score	13.3 ± 3.0	18.4 ± 1.9	<0.001
Activity Level	2.3 ± 0.7	3.6 ± 0.4	<0.001
Vigilance	2.4 ± 0.7	3.5 ± 0.6	<0.001
Productivity	2.7 ± 0.7	3.8 ± 0.3	<0.001
Intimacy & Sexual Relationship	3.0 ± 0.8	3.6 ± 0.6	<0.001
Social Outcome	2.8 ± 0.9	3.8 ± 0.4	<0.001
ESS Score	16.0 ± 4.6	7.7 ± 4.4	<0.001
PSQI Global Score	14.9 ± 7.1	10.4 ± 5.8	<0.001

Analyses are reported as mean ± SD. SSIS-Stigma and Social Impact Scale, HADS-Hospital Anxiety and Depression Scale, SF36—Short Form Health Survey, QOL- Quality of Life, FOSQ—Functional Outcomes of Sleep Questionnaire, ESS- Epworth Sleepiness Scale, PSQI-Pittsburgh Sleep Quality Index.

People with narcolepsy reported significantly more feelings of social rejection, financial Insecurity, internalized shame and social isolation than those without narcolepsy. They were more hesitant to disclose health information to others and were significantly below the norm in all domains of HRQOL, with the lowest HRQOL values in the social functioning and vitality domains. They reported being more anxious and depressed than controls, although in general anxiety and depression was mild in both groups. As expected, narcoleptics reported significantly more daytime sleepiness than controls. Both groups reported nighttime sleep disturbances beyond the norm, but narcoleptics reported lower nighttime sleep quality than controls.

Spearman correlation coefficients were computed to assess the relationship between the key variables in the narcoleptics. There were significant negative correlations between the total FOSQ score and all domains of health-related stigma (from internalized shame *r* = -0.212, *p* = 0.019 to social rejection *r* = -0.554, *p*<0.001), narcolepsy symptoms (*r* = -.419, *p*<0.001), anxiety (*r* = -.292, *p* = .001), depression (*r* = -0.585, *p* < 0.001), and nighttime sleep quality (*r* = -0.484, *p* < 0.001). There were significant positive correlations between the total FOSQ and vitality (*r* = 0.452, *p* < 0.001), educational status (*r* =. 223, *p* =. 001) and employment status (*r* = 0.174, *p* = 0.011).

We performed a hierarchical regression with the FOSQ total score as the dependent variable. The initial regression model included 4 blocks: demographic variables: age, educational status and employment status (block 1), psychosocial variables: anxiety and depression (block 2), narcolepsy-related variables: narcolepsy symptoms, vitality and nighttime sleep quality (block 3) and health-related stigma including the 4 stigma domains (block 4). The initial regression model accounted for 46.6% of the variance in the FOSQ with the psychosocial variables accounting for 25.6% beyond demographics, narcolepsy-related variables accounting for an additional 9.7% and stigma accounting for an additional 6.4% of the variance in the FOSQ. Least significant variables were individually systematically removed from the model. The final, best fitting model (Table 3) accounted for 45.7% of the variance in the FOSQ. In this model the most significant predictors of social functioning were depression (*p*<0.001), narcolepsy symptoms (*p* = 0.009) and social rejection (*p* = 0.001). Depression accounted for 34.9% of the variance in the FOSQ, narcolepsy symptoms accounted for 6.7% of the variance beyond depression and social rejection accounted for an additional 5.4% of the variance. Less depression, narcolepsy symptoms and perceived social rejection significantly predicting better functioning.

**Table 3:** Summary of the final hierarchical regression analysis predicting the FOSQ total score in narcoleptics (n = 122).

Variable	*B*	*SE B*	β	Adj. *R* ^*2*^	*P* Value
Step 1					
HADS Depression	-.403	.050	-.591	.344	<0.001
Step 2					
HADS Depression	-.365	.049	-.536	.407	<0.001
Narcolepsy Symptoms	-.013	.003	-.265		<0.001
Step 3					
HADS Depression	-.270	.054	-.395	.457	<0.001
Narcolepsy Symptoms	-.009	.003	-.191		0.009
Social Rejection	-.151	.043	-.289		0.001

HADS-Hospital Anxiety and Depression Scale.

**Fig 1:**
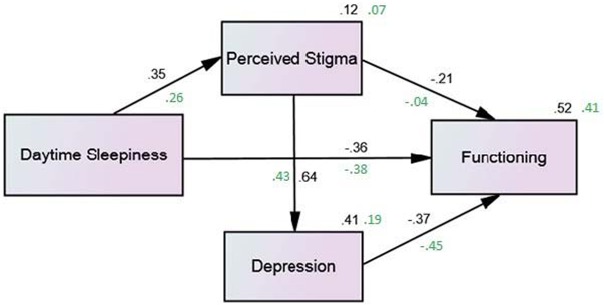
Path model: determinants of functioning in young adults with and without narcolepsy. Values: black = narcoleptics, green = controls. All of the paths in the final model were supported by the data (p<0.001) with the exception of the path from stigma to the FOSQ in the controls (p = 0.647). Fifty-two percent of the variance in functioning was explained by the final model in the narcoleptics and 41% was explained in the controls.

We performed path analyses using the variables in the final hierarchical model to assess the simultaneous relationships among variables separately in both groups. We substituted ESS for narcolepsy symptoms and substituted the sum of the stigma subscales for the individual subscales. The path models are depicted in Fig 1, and effects are reported in Table 4. All of the paths in the final model were supported by the data (*p*<0.001) with the exception of the path from stigma to the FOSQ in the controls (*p* = 0.647). Fifty-two percent of the variance in functioning was explained by the final model in the narcoleptics and 41% was explained in the controls. Fit indices for both models are presented in Table 5. An adequate fit of the data to the model is indicated by an RMSEA value less than. 08 and CFI greater than. 90. Results indicated a good model fit in the narcolepsy group and a model fit that while borderline, could be improved by removing the path from stigma to the FOSQ in the control group.

**Table 4:** Direct and indirect effects of key variables on functioning.

FOSQ^a^, Narcoleptics FOSQ^a^, Controls						
Variable	Direct	Indirect	Total	Direct	Indirect	Total
Sleepiness^b^	-.358	-.157	-.515	-.381	-.062	-.443
Stigma^c^	-.209	-.237	-.446	-.041	-.195	-.237
Depression^d^	-.372	-.000	-.372	-.450	. 000	-.450

Note. Effects are standardized,

^a^Functional Outcomes of Sleep total score,

^b^Epworth Sleepiness Scale,

^c^ Stigma and Social Impact Scale total score,

^d^HADS Depression.

**Table 5:** Path model fit indices.

	*X* ^2^	*df*	NFI	CFI	RMSEA
Narcoleptic	0.093	1	.999	1.000	.000
Control	1.659	1	.979	0.991	.085

Note. NFI = normed fit index, CFI = comparative fit index, RMSEA—root mean square error of approximation.

## Discussion and Conclusions

The findings of this study support the notion that young adults with narcolepsy are at risk for feeling stigmatized and that health-related stigma affects their functioning and HRQOL. First, we demonstrated that young adults with narcolepsy perceived significantly more stigma and lower mood and health-related quality of life than young adults without narcolepsy. Then we provided evidence to support the conclusion that health-related stigma likely affects their functioning directly and indirectly through depressed mood. We demonstrated that health-related stigma in young adults with narcolepsy is at a level consistent with health-related stigma in other chronic medical illnesses. To our knowledge, this is the first study focusing on stigma in narcolepsy. Young adults with narcolepsy reported relatively high levels of health-related stigma, significantly greater than controls without narcolepsy. Results are consistent with previous studies of health-related stigma in adults with other chronic illnesses such as epilepsy[31], multiple sclerosis[32] and HIV/AIDS[33]. In fact, we found health-related stigma levels in young adults with narcolepsy approximating those found in people with HIV by Fife and Wright[21] using the SSIS. They reported stigma levels (mean(SD)) of social rejection = 19.9(6), financial insecurity = 8.1(3), internalized shame = 13.7(3) and social isolation = 17.8(4) in people with HIV. In comparison, in our controls the levels were 10.7(3), 4.1(2), 7.0(3) and 4.1(2) respectively. The finding of high levels of health-related stigma in young adults with narcolepsy is important as there is growing evidence that stigma contributes to economic disparities and difficulties with social relationships, and can affect access to and the quality of health care as well as adherence to a medication regimen[3]. The observed association of health-related stigma, particularly social rejection, with functioning found in our analyses support findings in other chronic illnesses[34–36] and suggests that interventions addressing the stigma process could promote better functioning in young adults with narcolepsy.

Young adults with narcolepsy also reported lower health-related quality of life and greater anxiety and depression than young adults without narcolepsy. This is not surprising, and is in agreement with researchers who found that narcolepsy is associated with lower quality of life[7,11] and depression[37,38], especially in those with cataplexy[39]. Of concern is that the narcolepstics were particularly below the norm in role physical, vitality and social functioning, supporting findings previously reported by Daniels and colleagues[11]. Future research into and interventions to address these functional limitations in narcoleptics are indicated. We found that although on the whole, depression did not reach levels associated with clinical significance[40,41], it was directly related to lower functioning in both groups. However, 22% of the narcoleptics had depression scores greater than 10, suggesting clinically significant depression, while only 1% of the controls had depression scores greater than 10.

Results from this study are consistent with studies of young adults with Type 1 diabetes[42,43], epilepsy[44,45], HIV[46] that identified stigma as part of living with the disease and emphasized the impact of stigma on emotional health, social relationships and self-management of the illness. Findings will advance the field of sleep medicine by identifying that the young adult with narcolepsy may feel stigmatized and this can be negatively affecting their daily functioning and HRQOL. Now that this has been identified, many gaps remain. Research using qualitative methods may provide a richer understanding of health-related stigma from the perspective of the person with narcolepsy experiencing it. Future work is needed to characterize health-related stigma in middle age and older adults with narcolepsy. There is a need to develop and test strategies for prevention and management of stigmatization related to narcolepsy from the societal, organizational and individual perspective. Identifying people with narcolepsy at high risk for feeling stigmatized in order to implement preventive strategies is a promising area for future research. Studies of interventions for health-related stigma in HIV[47], mental illness[48,49] and epilepsy[50] have provided evidence to suggest that interventions using educational programs, skill-building, cognitive behavioral techniques and support groups may provide benefits.

Limitations of this research include the relatively small sample size, the smaller proportion of men in the narcolepsy group and the age of the data. In addition, the control group was largely recruited by participants with narcolepsy and this could have affected the results. However, one could expect that in this case less significant differences between groups would be seen. Finally, there may be other variables not included in our analyses that could affect functioning in young adults with narcolepsy. Besides the likelihood that this is the first published study of stigma in people with narcolepsy, strengths of this research include the use of well-established measures, a control group, and adequate sample size for the analyses.

In summary, our data suggest that health-related stigma is an important determinant of functioning in young adults with narcolepsy. Future work is indicated toward futher characterizing stigma and developing interventions that address various domains of stigma in people with narcolepsy.
